# The mitochondrial genome of a sea anemone *Bolocera* sp. exhibits novel genetic structures potentially involved in adaptation to the deep‐sea environment

**DOI:** 10.1002/ece3.3067

**Published:** 2017-05-30

**Authors:** Bo Zhang, Yan‐Hong Zhang, Xin Wang, Hui‐Xian Zhang, Qiang Lin

**Affiliations:** ^1^ CAS Key Laboratory of Tropical Marine Bio‐Resources and EcologySouth China Sea Institute of OceanologyChinese Academy of SciencesGuangzhouChina; ^2^ University of Chinese Academy of SciencesBeijingChina

**Keywords:** adaptation, *Bolocera* sp, deep‐sea sea anemone, mitochondrial genome

## Abstract

The deep sea is one of the most extensive ecosystems on earth. Organisms living there survive in an extremely harsh environment, and their mitochondrial energy metabolism might be a result of evolution. As one of the most important organelles, mitochondria generate energy through energy metabolism and play an important role in almost all biological activities. In this study, the mitogenome of a deep‐sea sea anemone (*Bolocera* sp.) was sequenced and characterized. Like other metazoans, it contained 13 energy pathway protein‐coding genes and two ribosomal RNAs. However, it also exhibited some unique features: just two transfer RNA genes, two group I introns, two transposon‐like noncanonical open reading frames (ORFs), and a control region‐like (CR‐like) element. All of the mitochondrial genes were coded by the same strand (the H‐strand). The genetic order and orientation were identical to those of most sequenced actiniarians. Phylogenetic analyses showed that this species was closely related to *Bolocera tuediae*. Positive selection analysis showed that three residues (31 L and 42 N in *ATP6*, 570 S in *ND5*) of *Bolocera* sp. were positively selected sites. By comparing these features with those of shallow sea anemone species, we deduced that these novel gene features may influence the activity of mitochondrial genes. This study may provide some clues regarding the adaptation of *Bolocera* sp. to the deep‐sea environment.

## 1 INTRODUCTION

The deep sea is the part of the ocean below the continental shelves, and it is the most extensive ecosystem on earth (Rex, 1981). The organisms living there survive in an extremely harsh environment, tolerating hundreds of bars of pressure, small amounts of oxygen, very little food, constant darkness, and low temperature (Sanders & Hessler, 1969). Because of the sparseness of animal life and the technical difficulties in sampling the deep‐sea benthos, our knowledge of deep‐sea organisms is based almost entirely on morphological distinctions at the species level (Etter, Rex, Chase, & Quattro, 1999). There is little information about the adaptive molecular mechanisms of the organisms living in the deep‐sea environment (Sanders & Hessler, 1969).

As the powerhouse of the cell, mitochondria generate energy by oxidative phosphorylation (OXPHOS) (Luo et al., 2008) and play important roles in energy metabolism and various biosynthetic pathways (Green & Reed, 1998; Newmeyer & Ferguson‐Miller, 2003). Mitochondria, with their own genetic material, are present in nearly all eukaryotic cells (Bernt, Braband, Schierwater, & Stadler, 2013), and they are autonomously replicated and transcribed (Boore, 1999). Mitogenomes have been widely used for studies of population genetics, phylogeography, phylogeny, and species identification (Brown, Brooke, Fordyce, & McCracken, 2011; Feng, Li, Kong, & Zheng, 2011; Gvoždík, Moravec, Klütsch, & Kotlik, 2010; Keskin & Can, 2009; Lei et al., 2010; Ma et al., 2013). The mitogenome, especially the 13 energy pathway protein‐coding genes, represents a particularly useful genetic marker for investigating the molecular basis of organismal adaptation to an extreme environment (Yu, Wang, Ting, & Zhang, 2011). In recent years, several mitochondrial genes have been shown to contain signatures of adaptive evolution, including the cytochrome b gene of alpacas (da Fonseca, Johnson, O'Brien, Ramos, & Antunes, 2008), the cytochrome c oxidase gene of plateau pikas (Luo et al., 2008), the NADH dehydrogenase six gene of domestic horses (Ning, Xiao, Li, Hua, & Zhang, 2010), and the ATP synthase genes of Caprinae (Hassanin, Ropiquet, Couloux, & Cruaud, 2009).

Most metazoan mitogenomes are circle molecules, between 14 and 18 kb in length that encode 37 genes (13 protein‐coding genes, 22 transfer RNA genes, and two ribosomal RNA genes) as well as a putative control region (Boore, 1999; Wolstenholme, 1992). As sea anemones are primitive animals (Putnam et al., 2007), their mitogenomes exhibit some differences. Similar to most metazoan mitogenomes, the typical sea anemone mitogenome is a circular molecule, 16–20 kb in length and encodes 13 energy pathway protein‐coding genes and two ribosomal RNA genes (Beagley, Okimoto, & Wolstenholme, 1998; Emblem et al., 2014; Osigus, Eitel, Bernt, Donath, & Schierwater, 2013). However, some distinctive features have been identified in these mitogenomes. Only 2 of the 22 essential transfer RNAs (tRNAs) are present, one or two genes are interrupted by a group I intron, the open reading frames (ORFs) encode unknown proteins, and there is a very slow nucleotide substitution rate (Beagley, Okada, & Wolstenholme, 1996; Beagley & Wolstenholme, 2013; Emblem et al., 2011; Johansen et al., 2010; Nielsen & Johansen, 2009; Osigus et al., 2013).

Although several complete or partial mitogenomes of sea anemones have been sequenced in recent years, the members of these genera are highly diverse (Emblem et al., 2014), and the information regarding these mitogenomes remains incomplete. For deep‐water species from extreme environments in particular, little mitochondrial data have been reported. To evaluate the variation in deep‐sea sea anemone gene structures and their adaptations to the deep‐sea environment, this study determined the complete mitogenome of a deep‐sea sea anemone (*Bolocera* sp.), identified its mitochondrial gene structures and arrangements, and elucidated their evolutionary characteristics.

## 2 MATERIALS AND METHODS

### 2.1 Specimens and DNA extraction

The specimen was collected on December 15, 2014, from a seamount on the Pacific Ocean (137.44˚E/8.52˚N) at a depth of 1106 m, fixed in 99% ethanol, and stored at 4°C. DNA was extracted using a Genomic DNA Kit (Tiangen Co. Beijing, China) according to the manufacturer's instructions. The specimen was not an endangered or protected species, and no specific permits were required for our collection process.

### 2.2 PCR and sequencing

To identify the subspecies of the specimen, two conserved genes (*COI* and *cytb*) were sequenced. The complete mitogenomes of closely related species were downloaded from the NCBI Entrez Database and amplified to search for conserved regions where primers for the complete mitogenome clone were designed. The complete mitogenome was amplified by overlapping PCR. All PCRs were performed in a 50 μl volume, which included 1 μl template DNA (approximately 100 ng), each primer at a concentration of 0.3 μmol/L, 5 μl of 10 × LA Taq buffer (Mg^2+^ plus), 5 μl of dNTP Mix (2.5 mmol/L), and 1 U of LA Taq (TaKaRa, Japan). The PCR amplifications used the following procedure: one cycle of denaturation for 5 min at 94°C; 35 cycles of 40 s at 94°C, 40 s at the primer‐specific annealing temperature, and 5 min at 72°C; and finally a 10‐min extension at 72°C. After purification, the PCR products were directly sequenced in both directions three times with the PCR primers. Sequencing was performed by ThermoFisher Scientific (Guangzhou, China).

### 2.3 Complete mitogenome analysis

The sequence alignment was conducted using Clustal X. The protein‐coding genes and rRNA genes were determined by BLAST and the NCBI Entrez Database and by comparison with the mitogenome sequences of homologous species. The tRNA genes and their secondary structures were predicted by the Web‐based tRNAscan‐SE 1.21 (Lowe & Eddy, 1997). The skew in nucleotide composition was calculated by AT skew and GC skew and measured according to the following formulae: AT skew = (*A* − *T*)/(*A* + *T*) and GC skew = (*G* − *C*)/(*G* + *C*) (Perna & Kocher, 1995), where *A*,* T*,* C*, and *G* are the occurrences of the corresponding bases. Codon usage was calculated by the Codon Usage Database (http://www.kazusa.or.jp/codon/). The gene map of the complete mitogenome was depicted by OGDRAW (http://ogdraw.mpimp-golm.mpg.de/).

### 2.4 Phylogenetic analysis

To illustrate the phylogenetic relationships among sea anemone, the complete mitogenomes of 23 Anthozoa species were downloaded from the GenBank database, including *Aiptasia pulchella* (HG423148), *Alicia sansibarensis* (KR051001), *Antholoba achates* (KR051002), *Bolocera tuediae* (HG423145), *Halcampoides purpurea* (KR051003), *Hormathia digitata* (HG423146), *Isosicyonis striata* (KR051006), *Metridium senile* (HG423143), *Nematostella* sp. (DQ643835), *Sagartia ornata* (KR051008), *Urticina eques* (HG423144), *Chrysopathes formosa* (NC_008411), *Myriopathes japonica* (NC_027667), *Stichopathes lutkeni* (NC_018377), *Savalia savaglia* (NC_008827), *Acropora aculeus* (KT001202), *Acropora tenuis* (NC003522), *Pocillopora damicornis* (EF526302), *Siderastrea radians* (DQ643838), *Heliopora coerulea* (NC_020375), *Dendronephthya suensoni* (NC_022809), *Renilla muelleri* (NC_018378), and *Stylatula elongata* (JX023275). *Geodia neptuni* (AY320032) (Demospongiae) was selected as the out‐group. The concatenated nucleotide sequences of 13 energy pathway protein‐coding genes were aligned using Clustal X with the default settings. The maximum likelihood (ML) method was employed to analyze the phylogenetic tree. The GTR + I + G model was selected as the best nucleotide substitution model by ModelTest 3.7 (Posada & Crandall, 1998). The ML analysis was performed by MEGA 5.1 with 1,000 bootstrap replicates.

### 2.5 Positive selection analysis

The selective pressure imposed on the mitogenomes of sea anemones was evaluated using CODEML from the PAML package. Two different tree‐building methods were used because the CODEML likelihood analysis is sensitive to tree topology. The two‐ratio and free‐ratio model (M1 model) was used in the mitogenome analysis. The branch‐site model was used to determine whether these genes have undergone positive selection in the foreground lineage. Bayes Empirical Bayes (BEB) analysis was used to calculate the Bayesian posterior probability of the positively selected sites.

## 3 RESULTS AND DISCUSSION

### 3.1 Genome organization

**Figure 1:**
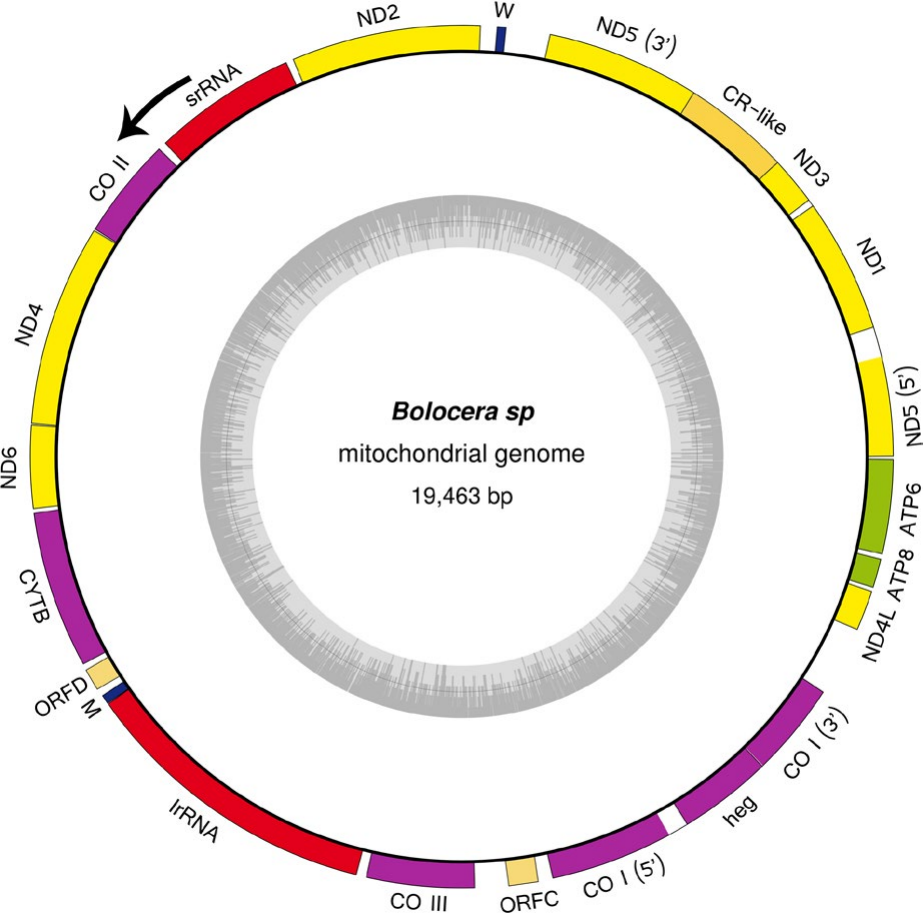
Graphical map of complete mitogenome of *Bolocera* sp. Different genes are represented by different boxes in different colors. tRNAs are displayed according to the one‐letter code. Genes encoded by the heavy strand are shown outside the circle, and those encoded by the light strand are shown inside. The direction of the arrows shows the direction of transcription. The inner ring shows the GC content of the mitogenome

Similar to most metazoan mitogenomes, the mitogenome of *Bolocera* sp. is a circular molecule. The complete mitochondrial DNA of *Bolocera* sp. contained 19,463 bp. It shared the highest overall similarity (96.43%) with the *B. tuediae* mitochondrial DNA sequence (Emblem et al., 2014). In addition to the common individual base composition differences, the alignment of the two complete mitogenomes also showed several insertions or deletions of genetic fragments. Therefore, we speculated that *Bolocera* sp. was a new subspecies of Bolocera. Similar to those of other anthozoan species, the mitogenome of *Bolocera* sp. showed a different evolution pattern than most metazoans (Shearer, Van Oppen, Romano, & Wörheide, 2002). It encoded 16 protein‐coding genes (13 energy pathway protein‐coding genes, a *heg* gene, and two unknown ORFs), two tRNA genes, and two rRNA genes (Figure 1, Table 1). All of the genes were coded by the same strand (the H‐strand) and transcribed in the same direction. The gene order and orientation were identical to other sequenced actiniarians. In all the mitogenomes studied, no gene overlaps were observed, and the intergenic spacers varied. Several typical anthozoan mitogenome features (Beagley & Wolstenholme, 2013; Beagley et al., 1998; Emblem et al., 2014) were also observed in *Bolocera* sp., including the presence of two group I introns, two tRNA genes, and large intergenic spacers. In addition, two noncanonical protein‐coding genes (ORFC and ORFD) were observed. A genetic fragment similar to the control region (CR) of metazoans was also observed, which was first reported in sea anemones. The complete mitochondrial DNA sequence was deposited in the GenBank database under the accession number KU507297.

**Table 1:** Gene structure of the mitogenome of *Bolocera* sp

Feature	Position numbers	Size		Codon		Intergenic nucleotides	Strand
		Nucleotides	Amino acid	Start	Stop		
*ND5*	1–717, 3,115–4,230	1,833	610	ATG	TAG	18	H
*ND1*	941–1,924	984	327	ATG	TAA	223	H
*ND3*	1,969–2,325	357	118	ATG	TAA	44	H
*tRNA* ^*Trp*^	4,544–4,613	70				313	H
*ND2*	4,729–6,111	1,383	460	ATG	TAG	115	H
*srRNA*	6,161–7,215	1,055				49	H
*COII*	7,269–8,015	747	248	ATG	TAA	53	H
*ND4*	8,021–9,496	1,476	491	ATG	TAA	5	H
*ND6*	9,501–10,109	609	202	ATG	TAA	4	H
*cytb*	10,137–11,288	1,152	383	ATG	TAG	27	H
ORFD	11,371–11,505	135	44	ATG	TAG	82	H
*tRNA* ^*Met*^	11,549–11,619	71				43	H
*lrRNA*	11,620–13,835	2,216				0	H
*COIII*	13,904–14,692	789	262	ATG	TAA	68	H
ORFC	14,946–15,146	201	66	ATG	TAG	253	H
*COI*	15,253–16,142, 16,996–17,677	1,572	523	ATG	TAA	106	H
*heg*	16,297–16,986	690	229	GTG	TAA	154	H
*ND4L*	18,182–18,481	300	99	ATG	TAA	504	H
*ATP8*	18,506–18,721	216	71	ATG	TAA	24	H
*ATP6*	18,755–19,444	690	229	ATG	TAA	33	H

### 3.2 Group I introns and unknown ORFs

**Figure 2:**
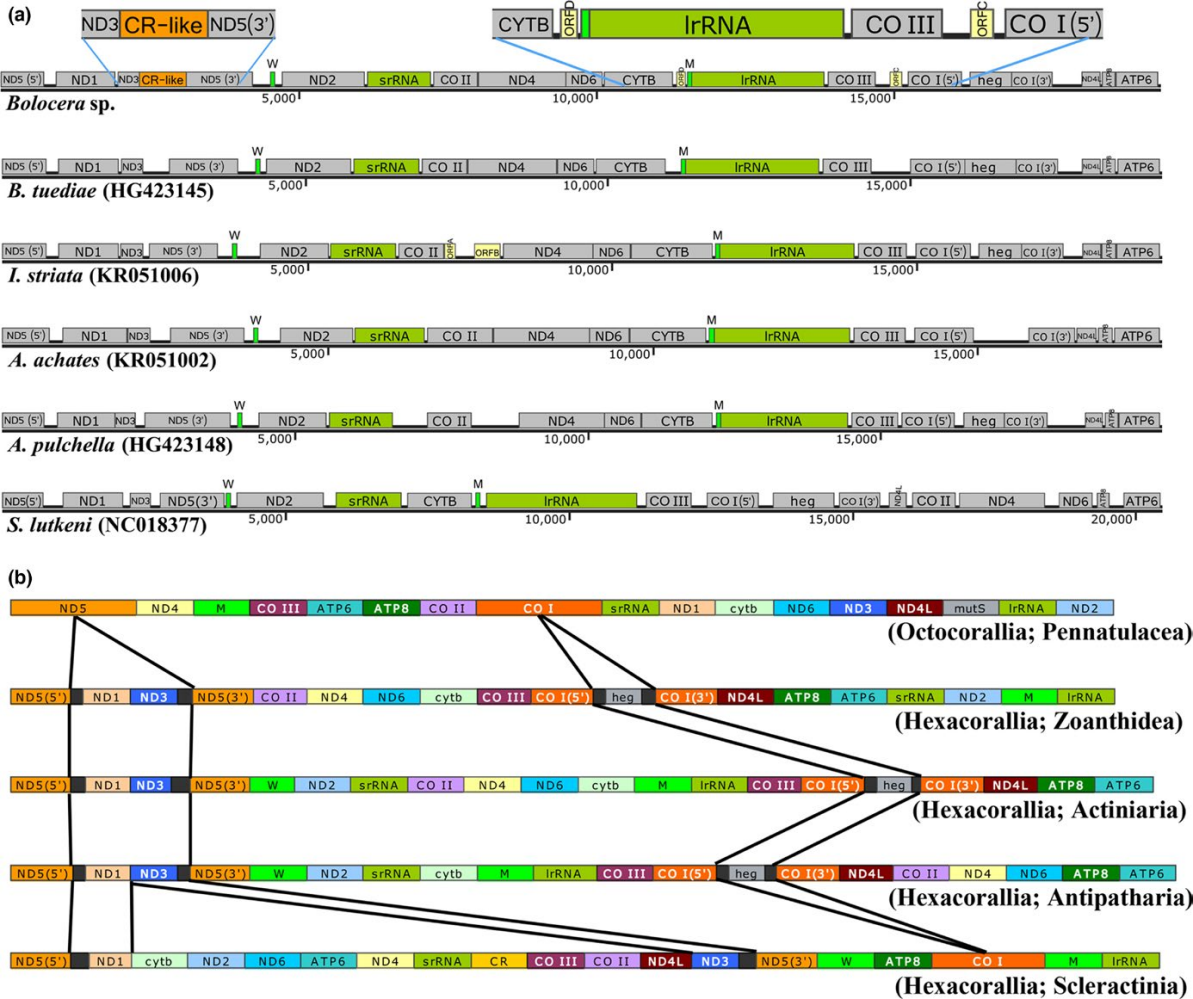
Linearized schemes of mitochondrial gene arrangements in anthozoans. (a) Linearized mitochondrial gene arrangements in actiniarians, *Stichopathes lutkeni* (Antipatharia) as outgroups. (b) Linearized mitochondrial gene arrangements in different suborders of anthozoa. Lengths of the genes correspond to relative lengths of the genomes in a. tRNAs are displayed according to the one‐letter code. Species names and NCBI accession numbers are given under each of the linearized schemes

In addition to the standard set of 13 energy pathway protein‐coding genes, an additional *heg* gene and two group I introns as well as two unknown ORFs were identified in the *Bolocera* sp. mitogenome (Figures 1 and 2, Table 1). Group I introns are genetic insertion elements that are extremely rare in metazoans and have only been identified within the mitogenomes of hexacorals and some sponges (Boore, 1999). In *Lophelia pertusa* (a scleractinian coral), a complex group I intron is inserted in the *ND5* gene to host seven essential mitochondrial protein genes and a rRNA gene (Emblem et al., 2011). In this study, two group I introns were detected (*ND5* intron and *CO I* intron). Both of the group I introns contained the same gene components as those in other hexacorallian species except for scleractinians (Figure 2). A gene structure analysis of hexacorallians showed that the *ND5* intron absorbed more genes but that the *CO I* intron lost the *heg* gene. The reason for this difference might be that the scleractinian ancestor had a different evolutionary pathway than other hexacorallian suborder species before significant mitogenome rearrangement, which is supported by the opinion of Mónica (Medina, Collins, Takaoka, Kuehl, & Boore, 2006).

Emblem et al. (2014) observed two ORFs (orfA‐51 and orfB‐26) of unknown function in the mitogenome of the cold‐water sea anemone *U. eques*, and a truncated version of orfA was also found in tropical reef *A. pulchella* and cold‐water *H. digitate* mitogenome. Foox, Brugler, Siddall, & Rodríguez (2016) also observed homologous ORFs in other actinioidean species, but no obvious similarity was noted within the ORFs. Instead of the ORFs described above, two new truncated ORFs (ORFC and ORFD) were observed in the *Bolocera* sp. mitogenome. Consistent with previous studies, no obvious similarity was detected. Flot & Tillier (2007) strongly suggested that the ORFs are expressed proteins, and Emblem even identified the ORFs to be functional elements (Emblem et al., 2014). The ORFs appear to be evolving under some selection relative to other protein‐coding genes (Emblem et al., 2014). The patterns (gained, lost, or truncated) exhibited by these noncanonical ORFs are consistent with transposon‐like elements (Winckler, Szafranski, & Glöckner, 2005). The ORFs detected in this study showed the same characteristics as the ORFs above, so they may play similar roles. Transposable elements can influence neighboring genes by altering splicing and polyadenylation patterns, or by functioning as enhancers or promoters (Girard & Freeling, 1999; Slotkin & Martienssen, 2007; Waterland & Jirtle, 2003). They have been demonstrated to play essential roles in the host response to stress and in facilitating the adaptation of populations (Blot, 1994; Casacuberta & González, 2013; Chénais, Caruso, Hiard, & Casse, 2012). Considering the common features of the species that carry noncanonical ORFs and the particular characteristics of the environment in which they live, the ORFs identified in this study may play important roles in adjusting mitochondrial energy metabolism.

### 3.3 Genome composition and skewness

In metazoan mitogenomes, the frequency of each nucleotide utilized varies among different taxa. The mitogenome of metazoans usually has a strand‐specific bias in nucleotide composition (Alexandre, Nelly, & Jean, 2005). The *A* + *T* content of the mitogenome is extremely high in insects and nematodes and lower in vertebrates (Saccone, Giorgi, Gissi, Pesole, & Reyes, 1999). In the anthozoan mitogenome, the *A* + *T* content ranges from 54.9% to 68.1% (Brugler & France, 2007; Foox et al., 2016). The nucleotide composition of the H‐strand in *Bolocera* sp. was biased toward *A* and *T*, and the overall *A* + *T* content was 60.51%. Similar results have also been observed in other actiniarian mitogenomes (Foox et al., 2016; Zhang & Zhu, 2016). As is commonly found in metazoan mitogenomes, the *A* + *T* content varied in different regions (Table 2). The CR‐like element presented the highest *A* + *T* content (66.16%), and the lowest content was found in the 2 tRNA genes (49.65%).

**Table 2:** Genomic characteristics of the mitogenome of *Bolocera* sp

Species	GenBank accession NO.	H‐strand				13 energy pathway protein‐coding genes^a^		lrRNA gene		srRNA gene		2 tRNA genes	
		Length (bp)	*A* + *T* (%)	AT‐skew	GC‐skew	NO. of amino acid	*A* + *T* (%)	Length (bp)	*A* + *T* (%)	Length (bp)	*A* + *T* (%)	Length (bp)	*A* + *T* (%)
*Bolocera* sp.	KU507297	19,463	60.51	−0.127	0.108	4,023	61.25	2,216	58.35	1,055	54.50	141	49.65
*Bolocera tuediae*	HG423145	19,143	60.28	−0.123	0.109	4,023	61.32	2,209	58.22	1,082	54.71	141	49.65
*Aiptasia pulchella*	HG423148	19,790	62.43	−0.104	0.110	4,090	62.76	2,178	60.42	1,065	57.00	141	47.52
*Alicia sansibarensis*	KR051001	19,575	61.01	−0.126	0.110	3,893	61.55	2,200	59.73	1,074	57.36	141	48.23
*Antholoba achates*	KR051002	17,816	61.89	−0.130	0.122	4,023	62.68	2,084	59.21	1,056	53.69	141	50.36
*Halcampoides purpurea*	KR051003	18,038	57.88	−0.108	0.083	3,933	58.67	2,192	57.48	1,082	55.27	141	51.06
*Hormathia digitata*	HG423146	18,754	61.79	−0.132	0.113	4,033	62.81	2,189	59.21	1,082	55.45	141	49.65
*Isosicyonis striata*	KR051006	19,001	60.28	−0.121	0.108	3,984	61.30	2,212	58.27	1,055	54.50	141	51.77
*Metridium senile*	HG423143	17,444	61.86	−0.129	0.112	3,953	62.67	2,188	59.37	1,082	55.27	141	51.06
*Nematostella* sp.	DQ643835	16,389	60.86	−0.117	0.090	3,945	61.42	602	56.81	693	57.29	141	49.65
*Sagartia ornata*	KR051008	17,446	62.21	−0.126	0.114	3,962	63.11	2,201	59.29	1,082	55.73	141	51.06
*Urticina eques*	HG423144	20,458	59.32	−0.118	0.094	3,956	60.47	2,214	57.50	1,057	53.93	141	49.65

a The *heg* gene and two ORFs do not counted in the 13 energy pathway protein‐coding genes.

The AT skew was negative (−0.127), whereas the GC skew was positive (0.108) in *Bolocera* sp. In all sequenced actiniarian mitogenomes, the trends were the same, and the AT‐skew and GC‐skew values were similar (Table 2). This result indicated that actiniarian mitogenomes favor Ts and Gs. Similar nucleotide skew patterns have also been observed in the mitogenomes of other hexacorallian subclasses (Brugler & France, 2007; van Oppen et al., 2000).

### 3.4 Protein‐coding genes

In this study, 16 protein‐coding genes (13 energy pathway protein‐coding genes, a *heg* gene, and two unknown ORFs) were identified (Table 1). All of these genes were coded by the same strand (the H‐strand) and transcribed in the same direction. In metazoans, most of the mitochondrial protein‐coding genes start with an ATN codon (Liao et al., 2010; Ma et al., 2014; Wang, Chao, Fang, & Yu, 2016). In the present study, except for the inserted *heg* gene, which is considered to be a selfish genetic element (Edgell, 2009), all of the genes were initiated by typical ATG codons. This pattern of initiation codon usage has also been observed in other basal metazoans (Beagley et al., 1998; Boore & Brown, 1995; Pont‐Kingdon et al., 1998).

In metazoan mitogenomes, stop codons are frequently incomplete (Clary & Wolstenholme, 1985; Okimoto, Macfarlane, & Wolstenholme, 1990) and are presumed to be completed by post transcriptional polyadenylation (Ojala, Montoya, & Attardi, 1981). However, in this study, all of the protein‐coding genes were terminated by complete TAA (11) and TAG (5) termination codons. This situation has also been observed in some sequenced cnidaria (Flot & Tillier, 2007; Kayal & Lavrov, 2008). The typical initiation codons and completed termination codons indicated a near standard genetic code, which is rare in metazoan mitogenomes. This result indicated that *Bolocera* sp. might retain some features of its cnidarian ancestor.

No significant difference in codon usage was detected among the sequenced actiniarians, and the *A* + *T* contents of the 13 energy pathway protein‐coding genes were 61.25% in *Bolocera* sp. In the 13 protein‐coding genes, UUA (Leu, 7.21%), UUU (Phe, 6.21%), and AUU (Ile, 4.56%) were the most frequently utilized codons in *Bolocera* sp., and the third position of the codons showed relatively high percentage of *A* and *T* bases (*A*, 26.58% and *T*, 38.06%). These features reflected codon usage with *A* and *T* biases at the third codon position, which is similar to the biases observed in most metazoans (Liao et al., 2010; Ma et al., 2014; Miller, Murphy, Burridge, & Austin, 2005; Wang et al., 2016).

### 3.5 Transfer RNA genes

**Figure 3:**
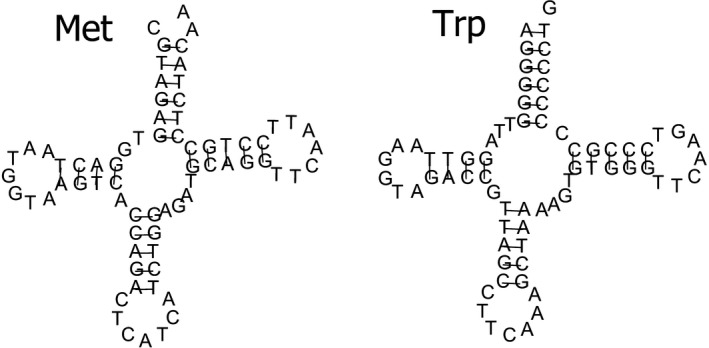
Inferred secondary structure of tRNAs in mitogenome of *Bolocera* sp.

In most cnidarian mitogenomes, only two tRNAs (tRNA^Trp^ and tRNA^Met^) were detected (except for octocorallians, in which only one tRNA^Met^ was detected) (Beagley et al., 1998; Beaton, Roge, & Cavalier‐Smith, 1998; Kayal & Lavrov, 2008). A study by Beagley and Wolstenholme (2013) showed that nuclear DNA‐encoded functional tRNAs were detected in mitochondria, and the missing tRNAs are believed to be encoded in the nucleus and later imported into the mitochondrion (Schneider & Maréchal‐Drouard, 2000). In this study, the mitogenome of *Bolocera* sp. contained two tRNAs (tRNA^Trp^ and tRNA^Met^) (Figure 3). Both tRNAs could fold into a clover‐leaf secondary structure, and the anticodon usage was identical to most of the observed sea anemone species. One mismatched base pair (C‐A) was detected in *tRNA* ^*Met*^. Interestingly, the unmatched base pair occurred on the amino acid acceptor arm. Such stem mismatches seem to be a common phenomenon for mitochondrial tRNAs in many species (Jiang et al., 2013; Liao et al., 2010; Miller et al., 2005; Wang et al., 2016) and are probably corrected by a post RNA‐editing mechanism (Lavrov, Brown, & Boore, 2000).

### 3.6 Noncoding regions

**Figure 4:**
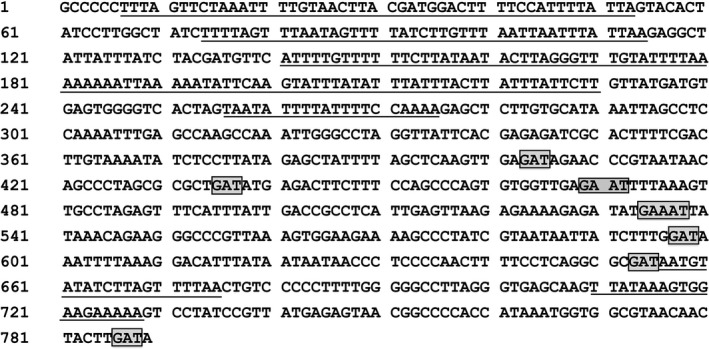
The CR‐like sequences of *Bolocera* sp. The *T* + *A*‐rich regions were underlined, and the “G(A)nT” motifs were marked with box

A total of 22 noncoding regions (ranging from 4 to 789 bp) were identified in the *Bolocera* sp. mitogenome. The longest noncoding region (789 bp) was located between the *ND3* gene and the second exon of the *ND5* gene. The nucleotide content of the 789‐bp noncoding region was 238 As (30.16%), 284 Ts (36.00%), 130 Cs (16.48%), and 137 Gs (17.36%). The *A* + *T* content (66.16%) of the 789‐bp noncoding region was higher than that of other regions in the mitogenome. In addition, several special TTTT and AAA repeats and *T* + *A*‐rich regions were observed (Figure 4).

**Figure 5:**
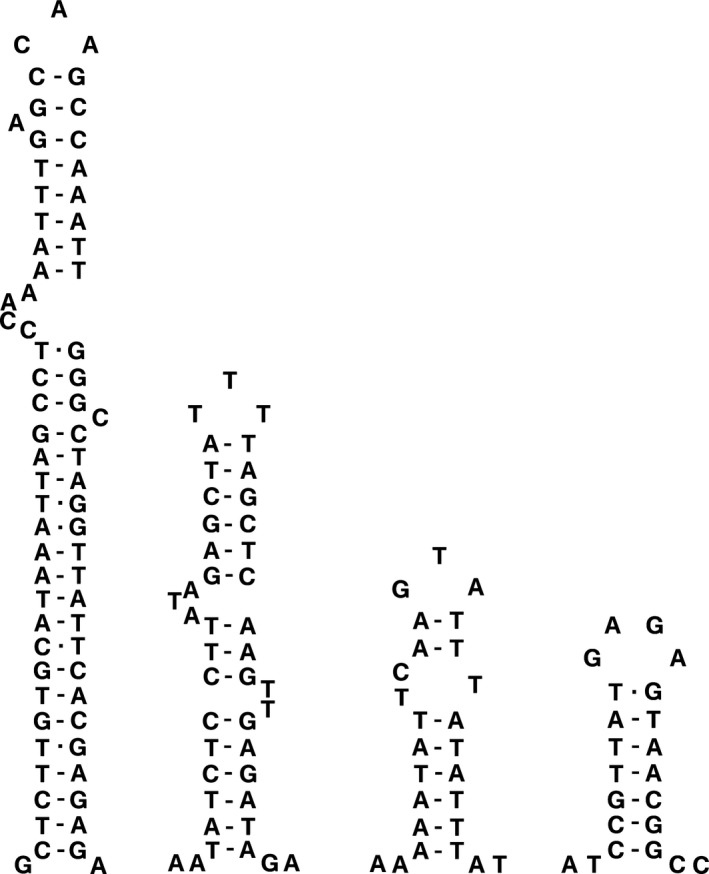
The potential stem‐loop structures in the 789‐bp noncoding “CR‐like” sequence of *Bolocera* sp.

The CR in the mitogenome is essential for transcription and replication in vertebrates (Fernández‐Silva, Enriquez, & Montoya, 2003). As it was not constrained in the same way as the protein‐coding genes, the mitochondrial CR is usually considered to be the most variable portion of the mitogenome (Marshall & Baker, 1997), showing the highest variation in the whole mitogenome (Aquadro & Greenberg, 1983). The structure of the CR has been intensively investigated in vertebrates, but not in invertebrates. Generally, in vertebrates, the mitochondrial CR is divided into three domains, including the extended terminal associated sequences (ETAS), the central conserved sequence blocks (CSB‐F, CSB‐E, and CSB‐D), and the conserved sequence blocks (CSB‐1, CSB‐2, and CSB‐3) (Pesole, Gissi, Chirico, & Saccone, 1999; Sbisà, Tanzariello, Reyes, Pesole, & Saccone, 1997). However, the nucleotide sequence, length, and number of each motif all vary considerably among vertebrate classes and even within a class (Rand, 1993; Ruokonen & Kvist, 2002; Shaffer & McKnight, 1996). In invertebrates, especially in anthozoans, similar CR structures are not clearly defined, but some similar features have been observed. In *A. tenuis*, the candidate mitochondrial CR contains repetitive elements and has the potential to form the typical secondary structures of vertebrate D–loops (van Oppen et al., 2000). In *Pocillopora*, the candidate mitochondrial CR exhibits three characteristics: large size, variability in nucleotide composition, and tandemly arranged repeated sequences (Flot & Tillier, 2007). With the exception of the repeated sequences, the rest of these characteristics were all observed in the 789‐bp noncoding region identified here. In addition, the special CR “G(A)nT” motif that is present in *S. gregaria* and *C. parallelus* (Zhang, Szymura, & Hewitt, 1995) was also observed in the 789‐bp noncoding region (Figure 4). In all organisms except primates, *A* + *T* > *G* + *C* in the CR domains (Sbisà et al., 1997), which was also observed in the 789‐bp noncoding region. Replication of mitogenome has been shown to initiate near hairpin structures (Clayton, 2000). In *Drosophila,* the origin of replication is located near a conserved stem‐loop structure (Saito, Tamura, & Aotsuka, 2005). In this study, the secondary structure of the 789‐bp noncoding fragment presented several similar stem‐loop structures (Figure 5), which is a characteristic feature of *O*_L_ in vertebrates (Clayton, 1991). In short, while no repetitive elements were observed, the 789‐bp noncoding region exhibited typical CR structures observed in other invertebrates. Considering the primitiveness of sea anemones in evolution, there must be some primitive features maintained. Therefore, we concluded that the 789‐bp noncoding sequence was a candidate CR (that we defined as “CR‐like”) that has not been reported in other actiniarians. This would be a unique and/or primitive mitogenomic feature for *Bolocera* sp., which is in agreement with the CR differences observed within invertebrates. As no other obvious adjusting elements were detected in the *Bolocera* sp. mitogenome, this special CR‐like element may play an important role in regulating the transcription and replication of the mitogenome in the extreme environment of the deep sea.

### 3.7 Phylogenetic analyses

**Figure 6:**
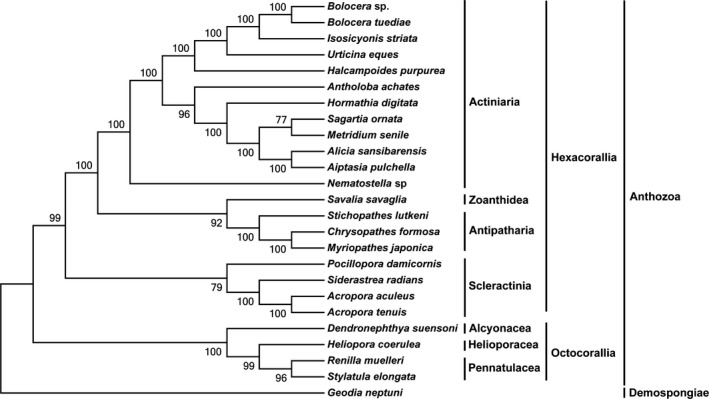
Phylogenetic tree of species of Anthozoa based on ML analysis of the nucleotide datasets. *Geodia neptuni* was selected as outgroup. Bootstrap support values are shown on the nodes

To investigate the relationships among anthozoan, we performed phylogenetic analysis based on the nucleotide datasets of the 13 mitochondrial energy pathway protein‐coding genes (Figure 6). All of the topologies showed a high support value. The phylogenetic tree of Anthozoa in this study showed that *Bolocera* sp. was clustered in the Actiniaria clade and had the closest relationship with *Bolocera tuediae*. The Actiniaria represented a monophyletic group and together with the sister groups Zoanthidea, Antipatharia, and Scleractinia clustered with the Hexacorallia group, which supported the classification of anthozoan (Daly, Fautin, & Cappola, 2003). Based on seven mitochondrial protein‐coding genes, Brugler and France (2007) found that zoanthid was at the base of the hexacoral clade, and the antipatharian clade had high support as a sister‐taxon to the scleractinian clade. However, in this study, based on the 13 protein‐coding genes, the antipatharians were a sister‐group to zoanthids, with a closer relationship to actiniarians, while scleractinians were located at the base of the hexacoral clade, which was identical to results obtained based on 18 S *rRNA* (Berntson, France, & Mullineaux, 1999). These findings also corroborated earlier studies of sea anemone phylogenetic relationships based on short mitochondrial and nucleotide sequences (Daly, Chaudhuri, Gusmão, & Rodríguez, 2008; Emblem et al., 2014).

### 3.8 Positive selection analysis

The selective pressures imposed on the mitogenomes of sea anemones were evaluated using CODEML from the PAML package (Table 3). Two different tree‐building methods were used because the CODEML likelihood analysis is sensitive to tree topology. There were no significantly different ω ratios between branches of genus *Bolocera* and other species when we set the genus *Bolocera* as a foreground branch using the two‐ratio model (*p* > .05). In the analyses of individual genes, we found that three positive selection sites (31 L and 42 N in *ATP6*, 570 S in *ND5*) showed BEB values >0.95 using branch‐site models.

**Table 3:** Selective pressure analyses of the mitochondrial genes of sea anemones

Gene	Branch‐site model							Model compared	2△ln*L*	LRT *p*‐value	Positive sites
	Model	ln *L*	Estimates of parameters								
*ATP6*	Model A	−3,368.2077	Site class	0.0000	1.0000	2a	2b	Model A versus Model A null	2.1423	.1433	31 L 0.994** 42 N 0.991**
			Proportion	0.0000	0.0000	0.9291	0.0709				
			Background ω	0.0719	1.0000	0.0719	1.0000				
			Foreground ω	0.0719	1.0000	999.0000	999.0000				
	Model A null	−3,369.2788									
*ND5*	Model A	−9,573.8530	Site class	0.0000	1.0000	2a	2b	Model A versus Model A null	0.3829	.5360	570 S 0.978*
			Proportion	0.0000	0.0000	0.9074	0.0926				
			Background ω	0.0815	1.0000	0.0815	1.0000				
			Foreground ω	0.0815	1.0000	26.8046	26.8046				
	Model A null	−9,574.0445									

Trees	Branch model			Model compared	2△ln *L*	LRT *p*‐value
	Model	ln *L*	Estimates of parameters			
NJ	Model 1	−58,898.9152		Model 1 versus Model 0	477.4924	.0000
	Two‐ratio	−59,137.6137	ω0=0.0968 ω1=0.0795	Two‐ratio versus Model 0	0.0954	.9534
	Model 0	−59,137.6614	ω=0.0968			
ML	Model 1	−58,898.6539		Model 1 versus Model 0	478.0150	.0000
	Two‐ratio	−59,137.6137	ω0=0.0968 ω1=0.0796	Two‐ratio versus Model 0	0.0954	.9534
	Model 0	−59,137.6614	ω=0.0968			

*posterior probability >95%; **posterior probability >99%.

The NADH dehydrogenase complex, which likely functions as a proton pump (da Fonseca et al., 2008) and influences metabolic performance (Hassanin et al., 2009), has been considered important in the adaptive evolution of the mammalian mitogenome (da Fonseca et al., 2008; Xu et al., 2007). *ND2* and *ND6* were found to be under positive selection pressure in a mitogenome analysis of Chinese snub‐nosed monkeys, which is suggestive of adaptive changes related to high altitude and cold weather stress (Yu et al., 2011). *ND6* was found to be under positive selection pressure in Tibetan horses living at high altitude (Ning et al., 2010). ATP synthase is directly associated with the produce of ATP (Mishmar et al., 2003; Weiss, Friedrich, Hofhaus, & Preis, 1991; Zhou, Shen, Irwin, Shen, & Zhang, 2014). It has been suggested that variation in ATPase proteins could result in significant variation in mitochondrial adaptation to different environments (Mishmar et al., 2003; Wallace, 2007). Members of the Caprini tribe that live in high‐altitude mountain regions show higher levels of adaptive evolution in the ATP synthase complex (Hassanin et al., 2009). The positive selection of *ATP6* and *ND5* observed in the present study could help us to better understand the adaptation of organisms to the deep‐sea environment.

## 4 CONCLUSION

This study characterized the complete mitogenome of a deep‐sea benthos species, *Bolocera* sp., which was speculated to be a new species of Bolocera. The study provided the following conclusions about deep‐sea organisms: (1) These organisms have monophyletic genome characteristics similar to those of shallow sea organisms: The basic gene content, order, and orientation of the species were identical to most of those reported homologous species, and phylogenetic analyses indicated that *Bolocera* sp. is closely related to *Bolocera tuediae* and belongs to the Actiniidae family. (2) Several genes experienced positive selection: Residues 31 L and 42 N in *ATP6* and 570 S in *ND5* were inferred to be positively selected sites for the branch of *Bolocera* sp. and *B. tuediae*, which may indicate that the genes were under positive selection pressure. (3) Novel genetic structures appeared: Some novel/unique gene features were observed in the mitogenomes of deep‐sea organisms compared with those of shallow sea species. In the mitogenome of *Bolocera* sp., two transposon‐like noncanonical ORFs and a CR‐like structure were detected. These novel genetic structures of *Bolocera* sp. may provide some clues regarding the adaptation to deep‐sea conditions. This study may shed light on the mitogenomic adaptation of sea anemones that inhabit the deep‐sea environment.

## CONFLICT OF INTEREST

The authors report no conflict of interests. The authors alone are responsible for the content and the writing of the article.
